# Retrieving Clinical Evidence: A Comparison of PubMed and Google Scholar for Quick Clinical Searches

**DOI:** 10.2196/jmir.2624

**Published:** 2013-08-15

**Authors:** Salimah Z Shariff, Shayna AD Bejaimal, Jessica M Sontrop, Arthur V Iansavichus, R Brian Haynes, Matthew A Weir, Amit X Garg

**Affiliations:** ^1^Kidney Clinical Research UnitDivision of NephrologyWestern UniversityLondon, ONCanada; ^2^Department of Epidemiology and BiostatisticsWestern UniversityLondon, ONCanada; ^3^McMaster UniversityDepartment of Clinical Epidemiology and BiostatisticsHamilton, ONCanada; ^4^Department of MedicineMcMaster UniversityHamilton, ONCanada

**Keywords:** information dissemination/methods, information storage and retrieval, medical, library science, PubMed, Google Scholar, nephrology

## Abstract

**Background:**

Physicians frequently search PubMed for information to guide patient care. More recently, Google Scholar has gained popularity as another freely accessible bibliographic database.

**Objective:**

To compare the performance of searches in PubMed and Google Scholar.

**Methods:**

We surveyed nephrologists (kidney specialists) and provided each with a unique clinical question derived from 100 renal therapy systematic reviews. Each physician provided the search terms they would type into a bibliographic database to locate evidence to answer the clinical question. We executed each of these searches in PubMed and Google Scholar and compared results for the first 40 records retrieved (equivalent to 2 default search pages in PubMed). We evaluated the recall (proportion of relevant articles found) and precision (ratio of relevant to nonrelevant articles) of the searches performed in PubMed and Google Scholar. Primary studies included in the systematic reviews served as the reference standard for relevant articles. We further documented whether relevant articles were available as free full-texts.

**Results:**

Compared with PubMed, the average search in Google Scholar retrieved twice as many relevant articles (PubMed: 11%; Google Scholar: 22%; *P*<.001). Precision was similar in both databases (PubMed: 6%; Google Scholar: 8%; *P*=.07). Google Scholar provided significantly greater access to free full-text publications (PubMed: 5%; Google Scholar: 14%; *P*<.001).

**Conclusions:**

For quick clinical searches, Google Scholar returns twice as many relevant articles as PubMed and provides greater access to free full-text articles.

## Introduction

With the explosion of available health information, physicians increasingly rely on bibliographic databases for health information to guide the care of their patients. Unfortunately, physicians face challenges when trying to find the information they need. They lack the time to develop efficient search strategies and often retrieve large numbers of nonrelevant articles [1-9]. Moreover, many bibliographic resources require paid subscriptions, which further limit access to current best evidence.

Two online resources that are freely accessible around the world are PubMed and Google Scholar. PubMed remains the most widely used resource for medical literature [10]. More recently, Google Scholar has gained popularity as an alternative online bibliographic search resource [11-21]. Available search features in Google Scholar and PubMed are contrasted in Table 1. Whereas PubMed indexes only peer reviewed biomedical literature, Google Scholar also indexes articles, theses, books, abstracts, and court opinions from a variety of disciplines and sources including academic publishers, professional societies, online repositories, universities, and other websites [22]. While PubMed orders articles in roughly reverse chronological order, Google Scholar aims to order articles by relevance using a proprietary algorithm that weighs information from the full text of each article, author, and journal information, and the number of times the article has been cited in other scholarly literature. Only a small fraction of the 21 million records in PubMed are available as free full-text publications via PubMed Central or specific journals. In contrast, due to its expanded search capabilities, Google Scholar may provide greater access to free full-text publications. To date, the utility of Google Scholar compared with PubMed for retrieving relevant primary literature to answer clinical questions has not been sufficiently tested.

In this study, we compare the ability of PubMed and Google Scholar to retrieve relevant renal literature for searches created by nephrologists to address questions of renal therapy. Renal literature is dispersed across more than 400 multidisciplinary journals and is difficult for nephrologists to track down; thus, this discipline provides an ideal model for this type of evaluation [23].

**Table 1:** Search features available in PubMed and Google Scholar.

Feature		PubMed	Google Scholar
**Searching**			
	Allows use of Boolean terms (AND, OR, NOT)	Yes	Yes
	Provides search limits (eg, age, publication type, date)	Yes (extensive)	Yes (very limited)
	Provides use of search filters that limit search results to a specific clinical study category or subject matter (eg, Clinical Queries, Topic-Specific Queries)	Yes	No
	Allows use of truncation (inclusion of multiple beginnings or endings achieved by typing in an asterisk “*” in PubMed – eg, cardio*)	Yes	No (automatically searches for variances in words)
	Allows use of controlled vocabulary (eg, MeSH terminology)	Yes	No
	Provides spell checking for search terms	Yes	Yes
	Stores search history	Yes	No
	Sorts results by relevance	No	Yes
**Access to articles**			
	Indicates whether articles are available as free full-texts	Yes	Yes
	Allows linking to institutions for subscription access (eg, link to university library)	Yes	Yes
**Other services**			
	Allows article citations to be imported into bibliography managers (eg, Reference Manager)	Yes (can import multiple selected citations)	Yes (can import only one citation at time)
	Tracks the number of times articles are cited by other publications	Yes (only to journals in PubMed Central)	Yes
	When searching, algorithm also searches the full-text of publications	No	Yes
	Provides email alerts for prespecified searches	Yes	Yes (introduced in 2010)
	Allows users to view related articles for an article of interest	Yes	Yes
	Source lists all journals and their publication dates that are included in their data holdings.	Yes	No

## Methods

### Clinical Questions and Articles of Interest

**Figure 1:**
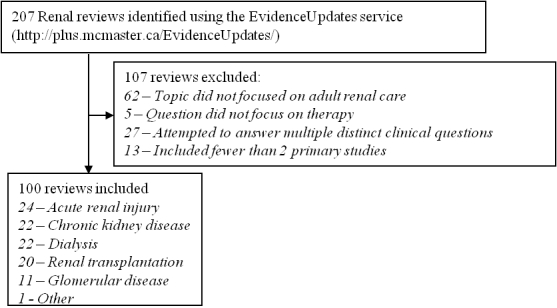
Process of selecting systematic reviews.

We derived 100 clinical questions from the objectives statements of 100 high-quality systematic reviews on renal therapy published between 2001 and 2009. We identified the systematic reviews from the EvidenceUpdates service in November 2009, by selecting the option to view all reviews for the discipline of nephrology; our search yielded 207 systematic reviews. This service prescreens and identifies systematic reviews and meta-analyses that meet strict methodological criteria and have a high potential for clinical relevance [24,25]. Two nephrologists used a previously developed checklist to independently confirm that each review targeted a single clinical question relevant to adult nephrology care (kappa=0.98) [26] and included at least 2 primary studies. Discrepancies were resolved through discussion; 100 reviews met the inclusion criteria (see Figure 1 for the process of selecting reviews). We transformed the objectives statement from each review into a clinical question (see Multimedia Appendix 1 for a sample of the questions posed and search queries received). For example, the objective of one review was “to assess the effectiveness of normal saline versus sodium bicarbonate for prevention of contrast-induced nephropathy”. We transformed this statement into the clinical question: “How effective is normal saline versus sodium bicarbonate for the prevention of contrast-induced nephropathy?”[27]. We extracted the citations to the primary studies referenced in each review that met the eligibility criteria for inclusion. These citations acted as a set of relevant articles for the corresponding clinical question (also referred to as the “reference standard”). The reviews cited a mean of 19 articles, totaling 1574 unique citations across all reviews.

### Data Collection and Measurements

We surveyed a simple random sample of nephrologists practicing in Canada (response rate 75%). Survey details are available elsewhere [28,29]. Briefly, we asked nephrologists about their information-gathering practices. In addition, we provided each nephrologist with a unique, randomly selected therapy-focused clinical question generated from a renal systematic review. The nephrologists provided the search terms they would type into a bibliographic resource to retrieve relevant studies to address the clinical question (known as a “search query”). The survey was designed and administered using the Dillman tailored design method [30]. The sampling frame consisted of nephrologists practicing in Canada and included both academic (practicing in a center with a fellowship training program) and community-based nephrologists. Nephrologists were selected from the sampling frame using a random number generator; one nephrologist was selected at a time and randomly assigned a clinical question. Once a selected nephrologist was deemed a nonresponder, the same clinical question was assigned to another nephrologist. In addition, upon receipt of a completed survey, if a respondent did not provide a search query to the clinical question, the same survey was re-administered to a new participant. Survey administration continued until 1 valid search query for each of the 100 questions was received. Overall, 115 survey responses were received and 15 were excluded from further analysis because of missing or illegible search queries (n=8) or because the survey was received after a physician was deemed a nonresponder (n=7).

To compare the performance of PubMed and Google Scholar for use by practicing physicians, we executed each physician-provided search query in PubMed and Google Scholar using all default settings. Occasionally, physicians provided misspelled search terms, acronyms, or other discrepancies. To address this, the syntax of the search was modified slightly using prespecified rules (listed in Multimedia Appendix 2). This was done in duplicate and differences were resolved by consensus. All searches were conducted between May and July 2010. We restricted each search to the search dates provided in the methods section of each systematic review. For each search result, we calculated the total number of records retrieved, the number of relevant articles retrieved, and the position of the relevant records in the search results. For each relevant article, we followed all links for full-text access and documented whether the full-text version could be retrieved for free. We did not use any pay-for-privilege accesses. To ensure that we did not inadvertently make use of our institution’s licensing when searching, all searches were conducted on a computer with Internet access provided by a local service provider and not our institution. We tested and validated our search methodology in a pilot phase. Two assessors (graduate students with expertise in computer science and biomedical science) independently conducted 10 searches in PubMed and Google Scholar and achieved a percent agreement of 99%.

### Content Coverage

To assess the potential for bias due to the absence of articles in one source over the other, we evaluated the content coverage for each database. A content coverage analysis determines whether pertinent literature is contained within a specific bibliographic database [31]. There are two potential reasons for not finding an important article when searching a database such as PubMed: either the article of interest is not included in the content holdings of the database (referred to as a lack of content coverage), or the article is present, but the search mechanism fails to retrieve it when a search phrase is typed into the database. To determine content coverage, we searched for each primary article using advanced search strategies as outlined in other coverage studies [32,33]. This involved various combinations of the manuscript’s title (both English and nonEnglish), the authors’ names, journal title, page numbers, and the year published. We selected all links to candidate matches to confirm a match. In Google Scholar, the option to view all versions for a candidate article was always selected and all links were attempted. If a primary article was not found in one of the resources, further searches were performed by another rater to confirm its absence. We previously published a more comprehensive content coverage analysis of renal literature that applied the same methods [34].

### General Statistical Analytic Strategy and Sample Size

#### Primary Analysis

The two most prominent performance metrics of searching are recall and precision (Table 2). Results from our survey indicated that 80% of nephrologists do not review beyond 40 search results, which is the equivalent of 2 default search pages in PubMed [28]. Thus, for the primary analysis, we calculated the recall and precision for the first 40 retrieved records in each search. We used a 2-sided paired *t* test to compare search outcomes between PubMed and Google Scholar. To reduce the risk of type I error, we used a conservative *P* value of .025 to interpret significance for all comparisons. We used SAS, Version 9.2 for all statistical analyses.

#### Secondary Analysis

We repeated the calculation for recall while only considering relevant articles that are freely accessible. For each physician-generated search, we also calculated the recall and precision for all retrieved records (not just the first 40).

**Table 2:** Formulas for calculating search recall^a^ and precision^b^ *.*

Search in PubMed or Google Scholar for a clinical question	Relevant articles^c^	Nonrelevant articles
Articles found	TP	FP
Articles not found	FN	TN

^a^Search recall TP/(TP + FN): the number of relevant articles found as a proportion of the total number of relevant articles.

^b^Search precision TP/(TP + FP) (also referred to as the positive predictive value in diagnostic test terminology): the number of relevant articles found as a proportion of the total number of articles found.

^c^For each search, the set of relevant articles were the collection primary studies included in the original systematic review from which the clinical question was derived.

## Results

### Nephrologist and Search Characteristics

Participating nephrologists were an average of 48 years old and had practiced nephrology for an average of 15 years. All respondents had used an online resource to guide the treatment of their patients in the previous year. Approximately 90% used PubMed to search, while 40% used Google Scholar; 32% indicated using both bibliographic resources. Searches provided by the nephrologists contained an average of three concept terms, with each term embodying a single concept, for example, myocardial infarction. Forty-eight percent of nephrologists used Boolean terms such as AND, OR, and NOT in their searches. Seven percent of searches included advanced search features such as search limits, search filters, and truncation (inclusion of multiple endings achieved by typing in an asterisk “*” in PubMed, eg, nephr*). No substantive differences were observed in searches provided by older versus younger nephrologists, males versus females, or by those practicing in an academic versus community setting.

### Content Coverage

PubMed and Google Scholar contained similar proportions of tested articles in their database holdings: each contained 78% of the 1574 unique citations collected. Google Scholar contained an additional 5% of the articles not included in PubMed and PubMed contained an additional 2% of the articles not included in Google Scholar; 15% of the articles were missing in both sources.

### Primary Analysis

Google Scholar retrieved twice as many relevant articles as PubMed within the first 40 records (average recall: 21.9% vs 10.9%; Table 3). Precision was similar in the two databases. When we considered both metrics together, Google Scholar demonstrated better recall and similar precision in 77% of searches.

### Secondary Analysis

Google Scholar retrieved three times as many relevant articles with free full-text access compared with PubMed (average recall: 15% vs 5%; *P*<0.001; Table 3). When examining all records (not just the first 40 records), PubMed and Google Scholar retrieved a similar number of relevant articles, although Google Scholar continued to provide increased free full-text access to publications. Overall, searches in Google Scholar retrieved more records per search compared with PubMed (median: 1000 records vs 148 records, respectively). This resulted in lower search precision in Google Scholar when all retrieved articles were considered.

**Table 3:** Recall and precision of physician searches tested in PubMed and Google Scholar (within the first 40 citations, PubMed found no relevant citations for 54% of the searches and Google Scholar found no relevant citations for 21% of the searches).

Measure^a^		Within first 40 citations		All citations	
		Mean, %^b^ (SD^c^)	*P* value^d^	Mean, %^b^ (SD^c^)	*P* value^d^
**Recall**			<.001		.10
	PubMed	10.9 (20)		38.0 (33)	
	Google Scholar	21.9 (24)		43.2 (29)	
**Free full-text recall**			<.001		<.001
	PubMed	4.7 (11)		16.4 (20)	
	Google Scholar	14.6 (20)		25.1 (23)	
**Precision**			.07		<.001
	PubMed	5.6 (11)		6.0 (11)	
	Google Scholar	7.6 (7)		0.8 (0.8)	

^a^Formulas for measures: (1) Recall: (number of relevant articles retrieved) / (total number of relevant articles available); (2) Free full-text recall: (number of relevant articles retrieved available for free full-text viewing) / (total number of relevant articles available), and (3) Precision: (number of relevant articles retrieved) / (total number of citations retrieved).

^b^Values represent mean of results from 100 searches.

^c^SD=Standard deviation.

^d^ *P* values compare PubMed to Google Scholar using a paired *t* test; significance values remained similar when using the nonparametric Wilcoxon signed rank test.

## Discussion

### Principal Findings

Nephrologists increasingly rely on online bibliographic databases to guide the care of their patients. Because most nephrologists view fewer than 40 search results, important literature will be missed if not contained within this narrow window [28]. For our primary objective, we compared the ability of PubMed and Google Scholar to retrieve relevant renal literature within the first 40 records. We found that Google Scholar retrieved twice as many relevant articles as PubMed—and three times as many relevant articles with free full-text access. These results are not attributable to differences in content coverage, as 78% of the tested articles were available in both databases [34]. Instead, the improved performance of Google Scholar may result from its method of ranking results based on relevance. However, when considering all search results (not just the first 40 records), recall was similar between Google Scholar and PubMed, while precision favored PubMed.

While many academics see the value in Google Scholar, its uptake has been slow among physicians [11-21,35-40]. Unlike Google Scholar, PubMed provides indexed content that is directly relevant to physicians, including clinical controlled vocabulary (MeSH [medical subject headings]), search limits (such as limiting articles by age or study type), and access to discipline-specific and methods search filters [24,41-43]. These advanced features have the potential to reduce the number of nonrelevant articles that are retrieved. However, only 7% of respondents used these features in their searches for this study. While 77% of nephrologists reported previous use of search limits, only 37% used controlled vocabularies, and only 20% used filters such as the Clinical Queries feature in PubMed [28,29]. Whereas PubMed searches retrieve published literature from biomedical journals, Google Scholar searches retrieve both published and unpublished literature from a range of disciplines. This may explain the greater overall number of records found per search (median of 1000 for Google Scholar and 148 for PubMed).

Google Scholar provided significantly greater access to free full-text articles. This is notable given that physicians and institutions in developing nations may lack the resources needed to maintain subscriptions to journals. Even in developed countries, the burden of paying for knowledge is felt. Some academic databases and journals have raised their fees for university subscriptions by up to 400%. This prompted one Canadian university library to cancel subscription access to the Web of Science bibliographic database, citing “a challenging fiscal climate” as a primary reason [44-46].

Our findings are consistent with those of previous studies [12,14,15,20,21]. In preliminary testing within targeted areas of respiratory care, sarcoma, pharmacotherapy, and family medicine, Google Scholar provided better comprehensiveness (recall) but worse efficiency (precision) compared with PubMed. Similar results were seen in our study when we considered all records that were retrieved and not just the first 40. However, previous studies tested only a small number of searches (range: 1-22), compared with the 100 searches in the current study. In addition, the search queries used in previous studies were created and tested by researchers in idealized settings, which may not generalize as well to searches generated by physicians in busy clinical settings.

We followed recommendations of search database evaluations from the field of information retrieval and designed our study to improve on limitations of previous studies [47,48]. To ensure that the clinical questions tested were relevant to practicing nephrologists, we gathered questions using renal systematic reviews that targeted questions in patient care where uncertainty exists. To ensure that all articles in the review were relevant for the clinical question, we selected systematic reviews that specified only one objective. Finally, to maximize external validity, we used a survey to obtain searches created by practicing nephrologists. Our survey achieved a response rate of 75% with responses from both newer and more seasoned nephrologists practicing in both community and academic settings [28].

### Limitations

Our study has some limitations. We did not directly observe the nephrologists as they searched. There may be a discrepancy between what search terms busy clinicians report in a survey and what they actually type in practice [37]. As recommended, we used primary studies included in high-quality systematic reviews to define relevance [14,20,49-54]. By using this method, we were unable to include other articles that some physicians may find relevant (eg, studies of lower methodological quality, narrative reviews, case reports, commentaries). However, our approach engages widely accepted principles of the hierarchy of evidence to identify the most important primary articles to retrieve in a search. For reasons of feasibility, our study focused on questions of therapy. As more systematic reviews for diagnosis, prognosis, and etiology are published, we will be able to expand this study to test searches for these types of studies as well. Our study tests the searches provided by the physicians, which are likely initial searches; however, in practice, an unsatisfactory search may be attempted again or modified based on the results obtained. Yet, our results indicate that Google Scholar can improve on the nephrologists’ initial search, which can save valuable clinical time. Given the nature of the survey, we are uncertain about how many steps nephrologists take to refine their search and future research should explore this. Although Google Scholar retrieved twice as many relevant articles as PubMed (in the first 40 citations), 80% of relevant articles were not retrieved by either source. Improved methods to efficiently retrieve relevant articles for clinical decision making requires further development and testing. Future research might also evaluate the effectiveness of strategies that apply relevance-based ranking to PubMed results on physician searches [55].

### Conclusions

In conclusion, for quick clinical searches, Google Scholar returns twice as many relevant articles as PubMed and provides greater access to free full-texts. Improved searching by clinicians has the potential to enhance the transfer of research into practice and improve patient care. Future studies should confirm these results for other medical disciplines.

## Multimedia Appendix 1

Sample of systematic reviews selected and search queries received by respondents.

## Multimedia Appendix 2

Rules used for syntactically improving physician searches in PubMed and Google Scholar.
