# Supplementary material for: Retrieving Clinical Evidence: A Comparison of PubMed and Google Scholar for Quick Clinical Searches
Source: J Med Internet Res. 2013 Aug 15;15(8):e164. doi: 10.2196/jmir.2624 (PMC3757915; doi:10.2196/jmir.2624)
Supplement: Supplementary file 1 [file jmir_v15i8e164_app1.pdf]

Multimedia Appendix 1. Sample of systematic reviews selected and search queries received by respondents.

**Selected Systematic Review Citation**	**Clinical Question Posed to Nephrologist**	**Search Query Response Received**
Landoni G, Biondi-Zoccai GG, Tumlin JA et al. Beneficial impact of fenoldopam in critically ill patients with or at risk for acute renal failure: a meta-analysis of randomized clinical trials. *Am J Kidney Dis* 2007;49(1):56-68.	What is the impact of fenoldopam on acute kidney injury, patient mortality, and length of hospital stay in critically ill patients?	fenoldapam and acute kidney injury
Douglas K, O'Malley PG, Jackson JL. Meta-analysis: the effect of statins on albuminuria. *Ann Intern Med* 2006;145(2):117-124.	Do statins affect albuminuria?	(albuminuria or microalbuminuria or proteinuria) and statin
MacKinnon M, Shurraw S, Akbari A, Knoll GA, Jaffey J, Clark HD. Combination therapy with an angiotensin receptor blocker and an ACE inhibitor in proteinuric renal disease: a systematic review of the efficacy and safety data. *Am J Kidney Dis* 2006;48(1):8-20.	What is the safety and efficacy of combination therapy with an ACE inhibitor and an ARB in patients with chronic proteinuric renal disease?	combination ace and arb therapy
Cruz DN, Perazella MA, Bellomo R et al. Extracorporeal blood purification therapies for prevention of radiocontrast-induced nephropathy: a systematic review. *Am J Kidney Dis* 2006;48(3):361-371.	Does periprocedural extracorporeal blood purification prevent radiocontrast-induced nephropathy?	blood purification contrast nephropathy
McCormack K, Rabindranath K, Kilonzo M et al. Systematic review of the effectiveness of preventing and treating Staphylococcus aureus carriage in reducing peritoneal catheter-related infections. *Health Technol Assess* 2007;11(23).	What is the clinical effectiveness of alternative strategies for the prevention and eradication of Staphylococcus aureus carriage in patients on peritoneal dialysis (PD)?	treatment staph aureus carriage and peritoneal dialysis
Ho KM, Sheridan DJ. Meta-analysis of frusemide to prevent or treat acute renal failure. *BMJ* 2006;333(7565):420.	What are the benefits and harms of frusemide in acute renal failure and do these effects differ when used to prevent or to treat acute renal failure?	utility of furosemide in acute renal failure
Garside R, Pitt M, Anderson R et al. The effectiveness and cost-effectiveness of cinacalcet for secondary hyperparathyroidism in end-stage renal disease patients on dialysis:	What is the efficacy of cinacalcet for the treatment of secondary hyperparathyroidism in people receiving chronic dialysis?	Hyperparathyroidism and its effects

a systematic review and economic evaluation. *Health Technol Assess* 2007;11(18).		
Jennings DL, Kalus JS, Coleman CI, Manierski C, Yee J. Combination therapy with an ACE inhibitor and an angiotensin receptor blocker for diabetic nephropathy: a meta-analysis. *Diabet Med* 2007;24(5):486-493.	Does combination renin–angiotensin–aldosterone system (RAAS)-inhibiting therapy provide greater benefit in diabetic nephropathy (DN) than angiotensin-converting enzyme inhibitor(s) (ACEIs) and angiotensin receptor blocker(s) (ARBs) therapy alone?	RAAS inhibitor and ACEI/ARBs
Rabindranath K, Adams J, Macleod AM, Muirhead N. Intermittent versus continuous renal replacement therapy for acute renal failure in adults. *Cochrane Database Syst Rev* 2007;(3).	Is intermittent hemodialysis or continuous renal replacement therapy superior in the treatment of acute renal failure (ARF)?	Acute ARF, Therapy, Hemodialysis, continuous
Phrommintikul A, Haas SJ, Elsik M, Krum H. Mortality and target haemoglobin concentrations in anaemic patients with chronic kidney disease treated with erythropoietin: a meta-analysis. *Lancet* 2007;369(9559):381-388.	In the treatment of anaemic chronic kidney disease patients with recombinant human erythropoietin, do different hemoglobin targets alter all-cause mortality or cardiovascular events?	hemoglobin targets chronic kidney disease mortality cardiovascular events anemia
