# Supplementary material for: Retrieving Clinical Evidence: A Comparison of PubMed and Google Scholar for Quick Clinical Searches
Source: J Med Internet Res. 2013 Aug 15;15(8):e164. doi: 10.2196/jmir.2624 (PMC3757915; doi:10.2196/jmir.2624)
Supplement: Supplementary file 2 [file jmir_v15i8e164_app2.pdf]

Multimedia Appendix 2. Rules used to syntactically improving physician searches in PubMed and Google Scholar

1. For PubMed Search: Update MeSH terms indicated in the query (exploded) and add PubMed syntax for limits described
   For Google Scholar Search: Convert MeSH terms indicated in the query into accepted terminology; for example, *"Kidney failure, Acute"[MeSH]* would be converted into *Acute kidney failure*
2. Correct spelling errors
3. Capitalize Boolean terms (AND, OR, NOT)
4. Remove stop words [adverbs, conjunctions, prepositions] (e.g. 'in', 'by', 'at', 'from', 'it', 'of', 'for')
5. Remove commas ',', periods '.' and semi-colons ';'
6. Replace '/' with OR
7. Replace 'and/or' with OR
8. Replace '+' with AND
9. Expand short forms or acronyms and include the original term with an OR; for example the search term *ckd* would be changed to *[(chronic kidney disease) OR ckd]*
